# Pulmonary arterial hypertension: Rationale for using multiple vs. single drug therapy

**DOI:** 10.21542/gcsp.2020.8

**Published:** 2020-04-30

**Authors:** Bradley A. Maron

**Affiliations:** 1 Department of Medicine, Division of Cardiovascular Medicine, Brigham and Women’s Hospital, Boston, MA, USA; 2 The Boston VA Healthcare System, West Roxbury, MA, USA

## Abstract

Pulmonary arterial hypertension (PAH) is defined by a heterogenous pathobiology that corresponds to variable clinical presentation, treatment response, and prognosis across affected patients. The approach to pharmacotherapeutics in PAH has evolved since the introduction of the first prostacyclin replacement drug, which was trialed in patients with end-stage disease as a strategy by which to delay or prevent mortality. Subsequently, the aim of care in PAH has shifted toward minimizing symptoms, improving functional capacity, delaying disease progression, and prolonging life. Thus, treatments are now implemented earlier and according to the evidence base, which spans more than twenty years and includes patients at various stages of disease. Overall, the evidence supports multidrug therapy rather than monotherapy in the majority of PAH patients. Among incident patients, up-front combination therapy with ambrisentan and tadalafil or other comparable agents within these drug classes is recommended based on strong clinical trial data. In the near future, up-front triple therapy may be emerge as *bona fide* treatment approach in selected patients. Future goals that are already under consideration in PAH include stronger integration of pathobiological characteristics when considering the use of specific drugs, or the development of novel therapies, toward precision medicine-based clinical pharmacology.

## Introduction

The proximate defining characteristic of pulmonary arterial hypertension (PAH) is a complex plexigenic arteriopathy that results in sub-total obliteration of distal arterioles.^1^ Hypertrophic, fibrotic, hypercontractile and proliferative changes occurring as a result of genetic predisposition, metabolic reprogramming, and post-transcriptional events among other processes promote arterial remodeling to increase pulmonary vascular resistance and, ultimately, right heart failure. These mechanistic underpinnings are integrated, and likely vary to some degree between patients and within the lung of individual patients.^2,3^ As a result, the PAH histopathophenotype and clinical presentation is heterogenous, adding an important measure of complexity to subclassifying patients and determining the optimal medical strategy for treatment.

Indeed, the initial treatment era of PAH focused on calcium channel antagonism, which improved symptoms (and in some cases could be considered curative) for subpopulations characterized by increased vasoreactivity as a central pathophysiology observed at point of care.^4^ This gave rise to one paradigm that emphasized monotherapy to treat PAH. In addition, the first wave of randomized trials included patients with end-stage PAH.^5^ This, in turn, focused attention on a subgroup of PAH that is particularly frail with marginal cardiopulmonary reserve and for whom the safety of expanding medical therapy was not known or viewed by many as laden with risk.

Thus, reconciling the heterogeneous and integrated pathobiological basis of PAH with lingering concern regarding efficacy and safety of multidrug therapeutic approaches emerged as an early, but defining dilemma in the field. An overview of this topic was presented recently at the Sir Magdi Yacoub Aswan Heart Center Science and Practice Series (Aswan, Egypt), as part of the Pulmonary Vascular Research Institute’s commitment to global education.^6^ The current work reviews the rationale for using single vs. multiple drug therapy to treat PAH, which was the central topic of that presentation.

## Rationale for multi-drug therapy in PAH

A strong precedent exists in support of diversifying drug therapy to manage a wide spectrum of diseases, including highly prevalent cardiopulmonary disorders such as systemic hypertension, atrial fibrillation, myocardial infarction and others (Table 1).^7^

**Table 1:** Common disease that often require multidrug therapy. COPD, chronic obstructive pulmonary disease; HIV, human immunodeficiency syndrome. Concept adapted from Ref. [7] and through personal communication with Dr. Evan Brittain.

Common diseases requiring ≥2 pharmacotherapies	
Systemic Hypertension	Asthma
Congestive Heart Failure	Hypercholesterolemia
COPD	Myocardial Infarction
Atrial Fibrillation	Systemic Sclerosis
Rheumatoid arthritis	Depression
Diabetes Mellitus	Schizophrenia
HIV	Gout
Cancer	Sepsis

In forms of pulmonary hypertension (PH) other than PAH (e.g., PH due to left-heart disease or lung disease), there is an unfortunate trend toward including unproven medical therapies for the routine management of patients.^8^ Since this approach may be associated with adverse clinical events, it is important to confine decision-making to evidence-based strategies.

**Figure 1.**
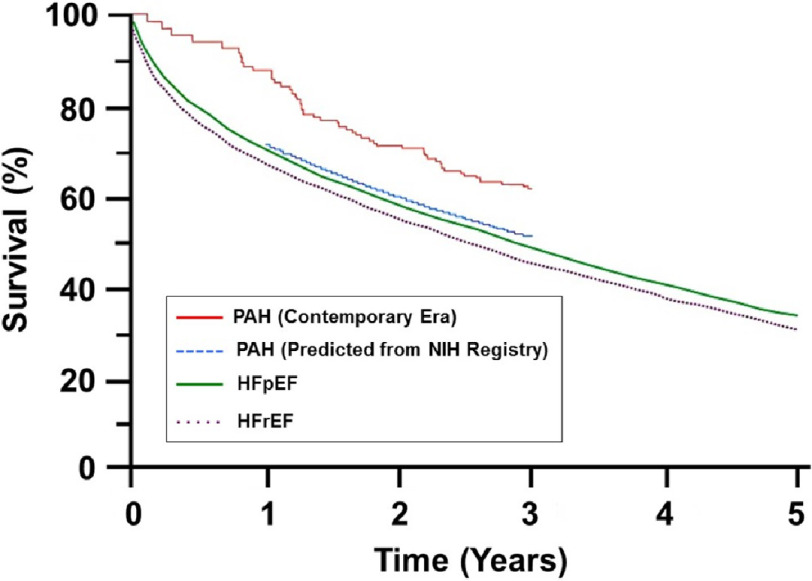
Mortality rates in patients with pulmonary arterial hypertension (PAH), left-sided heart failure with reduced ejection fraction (HFrEF), and left-sided heart failure with preserved ejection fraction (HFpEF). An extrapolated Kaplan–Meier analyses converging different data sets to compare mortality rates from HFpEF (purple dotted line), HFrEF (green solid line), and PAH (observed, blue dotted line; predicted, red line). *Adapted with permission from Ref.* [9].

In PAH, this is possible through the accumulation of clinical trial data spanning more than two decades.^1,5^ It is, in fact, by virtue of persistent effort among the clinical trialist community that has steadily improved PAH outcome compared to the original era in which disease-specific therapies were lacking. Comparative analysis of epidemiological data suggest that outcome in PAH is now more favorable than for patients with other, far more common cardiovascular disorders including various forms of left heart failure (Figure 1).^9^

The pathobiological framework of PAH is wide, implicating numerous different molecular mechanisms that regulate pathogenic vascular remodeling or modulate right ventricular dysfunction directly through afterload-independent pathways. It follows that new potential drug therapies continue to emerge frequently and are being tested in Phase I-III clinical trials at an expeditious rate (as reviewed in Ref. [10]). At present, however, there are 14 U.S. Food and Drug Administration (FDA)-approved medical therapies for PAH, all of which affect the same three targets: nitric oxide (NO⋅) signaling (soluble guanylyl cyclase stimulator and phosphodiesterase type-V inhibitor [PDE-Vi] classes), the endothelin (ET) receptor axis (selective ET_A_ and non-selective ET_A∕B_ antagonists; collectively referred to as endothelin receptor antagonists [ERAs]), and the prostacyclin pathways (prostaglandin-I_2_ replacement or counter-receptor agonism).^1^ Thus, the totality of data on multi-drug therapy centers is around the implementation of these therapies used in combination sequentially or combined up-front in the management of patients.

## Evidence base for multidrug therapy in PAH

Intravenous epoprostenol (PGI_2_), reserved in practice today as an initial treatment for use in patients with particularly elevated clinical risk, was the only available pharmacotherapy in PAH from 1995 until access to the non-selective ET_A∕B_ receptor antagonist bosentan became available in 2001.

In 2004, Humbert and colleagues completed the first double-blind, placebo-controlled study testing the effect of add-on therapy to prostacyclin.^11^ Patients were started on epoprostenol (2 ng/kg/min to maximum 14 ng/kg/min at week 16) and then randomized to receive bosentan 62.5 mg b.i.d. for 4 weeks followed by 125 mg po b.i.d. or placebo. Compared to placebo, the median decrease in pulmonary vascular resistance in the bosentan arm was significantly greater than placebo (-8.1 WU vs. -2.4 WU). Later, it was determined that the addition of the inhaled prostacyclin analogue, iloprost, to stable monotherapy with bosentan was similarly effective in patients with New York Heart Association Functional Class (NYHA-FC) III symptoms but in whom parenteral epoprostenol was not indicated or was contraindicated.^12^

The wider availability and favorable safety profile of PDE-Vi drugs made add-on treatment with this class an important consideration, and one that could be tested in a larger patient population than preceding studies. Simonneau and colleagues completed a multinational study including 41 centers in 11 countries over 3 years and enrolling N=267 patients with idiopathic, associated anorexigen use or connective tissue disease, or congenital PAH.^13^ A placebo-adjusted increase in the 6-minute walk distance (6-MWD) by +28.8 m was observed in patients randomized to sildenafil (titrated to a maximum does of 80 mg t.i.d.) compared to +1.0 m for placebo. The benefit of add-on therapy was particularly notable among patients entering the study with a higher 6-MWD, implying that earlier intervention may be associated with an optimal treatment response. The salutary benefit of add-on therapy in this study also included improvement in central cardiopulmonary hemodynamics as well as longer time to clinical worsening.

## Initial assessments of dual PAH therapy on clinical response in real-world settings

Clinical trials in PAH have greatly improved patient outcome and should be a benchmark for aptitude in the advancement of other uncommon diseases. However, trial enrollment criteria is particularly stringent in PAH owing to clinical and pathobiological heterogeneity, and, therefore, it is imperative to understand the extent to which treatment paradigms affect patients in the ‘real world’ setting.^14^

In one retrospective study, the effect of sildenafil add-on to bosentan or *vice versa* on key clinical metrics was studied in a cohort of N=93 consecutive idiopathic or hereditary PAH patients evaluated at the National Referral Center in Bologna, Italy.^15^ The investigators included patients for whom sequential therapy was implemented due to failure for patients to meet pre-specified treatment goals on monotherapy (e.g., NYHA FC I/II; cardiac index ≥2.5 L/min/m^2^ and right atrial pressure <10 mmHg). An assessment including 6-MWD, right heart catheterization, and NYHA-FC recording was performed at the time of treatment change and 3-4 months thereafter. Compared to baseline, data collected at the end of the follow-up period showed that significant fewer idiopathic or hereditary PAH patients (N=93) had NYHA-FC III/IV (51 vs. 32), increased 6-MWD by a median of +48 m, and improved cardiopulmonary hemodynamics including cardiac index +0.4 L/min/m^2^ and PVR -2.0 WU.

**Figure 2.**
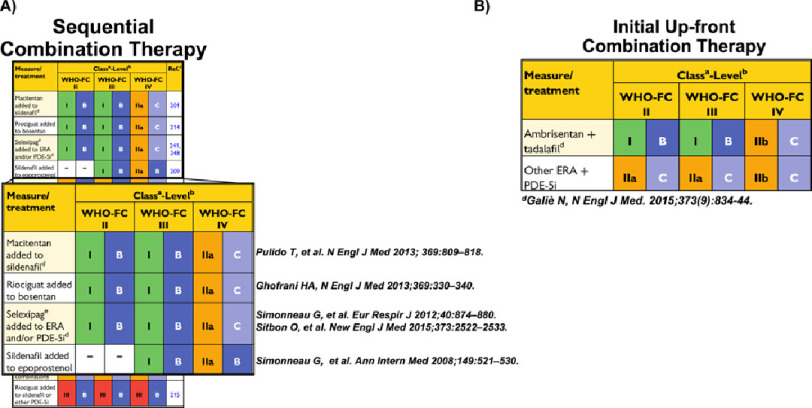
Selected recommendations for efficacy of (A) sequential add-on and (B) initial drug combination therapy for pulmonary arterial hypertension according to World Health Organization Functional Class. Specific references in support of sub-recommendations are provided as insets. ERA, endothelin receptor antagonist; PDE-5i, phosphodiesterase type-5 inhibitor; WHO-FC, World Health Organization Functional Class. *Adapted with permission from Ref.* [16].

Based on post-hoc analyses in PAH randomized clinical trials, sequential add-on therapy is now a Class IB (Level of Evidence B) European Respiratory Society/European Society of Cardiology (ERS/ESC) Guideline recommendation^16^ in patients with mild or moderate symptom burden for macitentan (non-selective ET_A∕B_ antagonist) added to sildenafil; riociguat (sGC stimulator) added to bosentan; selexipag (prostacyclin receptor agonist) added to any endothelin receptor antagonist or PDE-Vi therapy (Figure 2A). A comprehensive list of potential combination therapies is detailed in Ref. [16]. Owing to augmentation of NO-dependent vasodilation on hemodynamic stability, PDE-Vi drugs should not be used in combination with riociguat.

## Combination up-front therapy in treatment-naïve PAH patients

The concept of combination up-front, as opposed to sequential add-on therapy in PAH was modeled conceptually after such a strategy was implemented successfully in other complex diseases, such as human immunodeficiency virus.^17^ To that end, the Ambrisentan and Tadalafil in Patients with Pulmonary Arterial Hypertension (AMBITION) trial was a landmark multi-center international study that enrolled N=500 treatment-naïve PAH patients within about 10 days of diagnosis.^18^

This study was innovative on many levels, including the end-point itself, which definitively transitioned away from single dimensional assessments in favor of comprehensive yet disease-specific measures. Specifically, the primary end-point was based on a time-to-event analysis driven by the first event of clinical failure, which was defined as the first occurrence of a composite of death, hospitalization for worsening pulmonary arterial hypertension, disease progression, or unsatisfactory long-term clinical response.

Patients were randomized using a 2:1:1 strategy to receive combination tadalafil (PDE-Vi) and ambrisentan (ET_A_ antagonist) vs. monotherapy with tadalafil vs. monotherapy with ambrisentan. The hazard ratio of the primary end-point for the patients randomized to the combination treatment arm vs. monotherapy with either tadalafil or ambrisentan was 0.50 (95% confidence interval 0.35–0.72, *P* < 0.001), and at week 24 of the study there was a greater reduction in biochemical evidence of heart failure measured using N-terminal pro-brain natriuretic peptide (NT-BNP) level (mean Δ -67.2% vs. -50.4%, *P* < 0.0001) and a higher rate of satisfactory clinic response (39% vs. 29%).

Although an intricate strategy was utilized to safely achieve the target dose of both drugs (spanning 8 weeks), major side effects were not observed at a prohibitive rate in the combination therapy group. Furthermore, the clinical benefits was internally consistent across nearly all subgroups. To avoid overemphasizing the utility of this specific drug therapy combination, the ERS/ESC Guidelines categorize the use of any ERA plus PDE-Vi as a Class IIb (Level of Evidence C) indication for up-front treatment of incident PAH patients (Figure 2B).^16^ Alternative drug combinations achieving Class IIa or IIb Recommendation, but are less well-defined for World Health Organization-FC II patients are outlined in detail in Ref. [16].

## Future directions

Escalation in the approach to PAH medical therapy would seem to drive gains in mortality reported observed in large and validated registries. For example, the 1- and 3-year mortality rate in the Registry to Evaluate Early And Long-term PAH Disease Management (REVAL)^19^ and French^20^ registries prior to mainstream utilization of combination therapy were 85% and 87%, respectively, and 68% and 67%, respectively. However, in a contemporary analysis including 97 patients with newly diagnosed PAH treated with initial dual oral treatment of various combinations (86% had NYHA FC III/IV), Sitbon and colleagues^21^ reported 1- and 3-year survival rates that were substantially higher: 97% and 94%, respectively. Emerging data in carefully selected PAH patients suggests that up-front triple therapy with an ERA, PDE-Vi, and prostacyclin modulator is highly effective, and this topic is reviewed in greater detail elsewhere in this issue of the journal by Professor Simonneau.

Notwithstanding these favorable trends in populations, many individual patients remain treatment non-responders, and the burden on patient quality of life and healthcare economics remains staggering.^22^ Taken together, a strong incentive remains to improve knowledge on pathophysiology, clinical trial design and rationale drug therapy selection in PAH patients. Specifically, there is increasing attention on using Mendelian randomization,^23^ biomarker-driven,^24^ and network medicine-based approaches to optimize the pathobiology-drug target relationship for clinical trial participants.^25^ It may be the case the future of clinical trial enrollment in the era of precision medicine includes alternative study designs, such as N-of-1 trials,^9^ to allow better insight into strategies that tailor drug selection based on the unique and individualized pathobiological profile.

**Figure 3.**
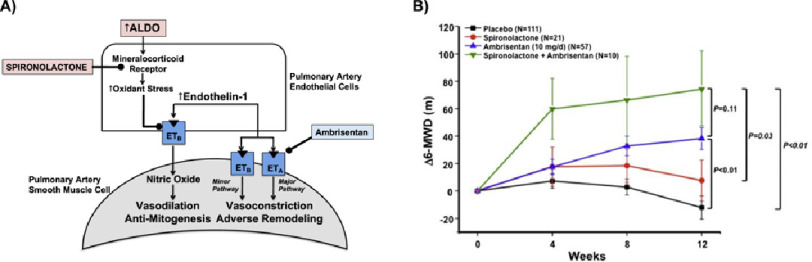
Biological hypothesis for repurposing spironolactone therapy and proof-of-concept supportive clinical data. (A) Schematic representation of the biological hypothesis supporting combination spironolactone (SPIRO) plus endothelin type-A receptor (ET_A_) antagonism for the treatment of PAH. Stimulation of pulmonary artery smooth muscle cells (PASMC) ET_A_ and endothelin type-B receptor (ET_B_) are the major and minor signaling pathways, respectively, that modulate endothelin-1 (ET-1)-dependent pulmonary vasoconstriction in PAH. In contrast, pulmonary artery endothelial cell (PAEC) ET_B_ stimulation by ET-1 promotes pulmonary vasodilation. In PAH, hyperaldosteronism is associated with a pulmonary vasculopathy that is due, in part, to increased oxidant stress levels that inhibits ET_B_ function. Therefore, two potential treatment targets within the aldosterone-endothelin receptor axis are exposed: ET_A_ to inhibit the major pulmonary vasoconstrictor pathway and the mineralocorticoid receptor (MR) to preserve ET_B_-dependent vasodilation. (B) A retrospective analysis of ARIES-1 and -2 trials^30^ were performed. Change from baseline in the 6-MWD. Mean ± SE change from baseline in the 6-MWD at week 4, 8, and 12 in the placebo (N=111), spironolactone (N=21), ambrisentan (10 mg/day, N=57), and ambrisentan + spironolactone (N=10) treatment groups. P-Values reflect comparisons among groups at week 12. *Reproduced with permission from Ref*. ^31^.

Along these lines, there is substantial interest in drug repurposing as one major strategy by which to increase availability of potential effective drugs with known side-effect profiles.^26^ The selection an agent could be based on *in silico* screening tools, or empiric evidence. For example, the mineralocorticoid hormone aldosterone (ALDO) is increased in PAH and associates positively with NYHA FC status.^27,28^ We have demonstrated that increased ALDO oxidizes the ET_B_ receptor in pulmonary endothelial cells to limit ET_B_-dependent nitric oxide synthesis and promote vascular fibrosis.^29^ Based on these empiric data, we hypothesized that ALDO antagonism with spironolactone in combination with ambrisentan would be an potential favorable drug combination by leveraging the anti-fibrotic effect of spironolactone with the anti-contractile and anti-mitogenic effect of ET_A_-receptor antagonism in vascular smooth muscle cells (Figure 3).

Retrospective analysis of the ARIES-1 and -2 trials,^30^ which were randomized placebo-controlled clinical studies testing the therapeutic effect of ambrisentan over 12 weeks in PAH yielded results in support of this proof-of-concept.^31^ Among participants randomized to ambrisentan 10 mg/d, patients using spironolactone (N=21) as part of their standard of care medical regimen had a trend toward improved 6-MWD by 94% at week 12, improved plasma BNP concentration by 1.7-fold, and improved WHO FC status compared to patients not taking spironolactone (N=10). These data were limited by low patient volume among other reasons, but nonetheless provides one example by which drug repurposing may be validated through experimental and existing datasets. Other examples of repurposing with promising intermediate data include histamine receptor antagonists, angiotensin converting enzyme (ACE) axis modulators, and metformin.^32,33^

## Conclusions

There is now ample evidence to support a multi-drug approach to the treatment of PAH, which includes up-front combination therapy in treatment-naïve patients using ambrisentan and tadalafil or other ERA and PDE-Vi. The evolution in treatment strategy from monotherapy in the prior generation parallels an emphasis on earlier detection of patients in advance of end-stage disease. In this way, patients are generally characterized by more favorable cardiopulmonary reserve, less fragility, and wider tolerance of side effects that allows for maximal clinical benefit of therapeutic intervention. Up-front triple therapy is on the horizon, as more data using this approach becomes available. Cutting-edge clinical trial enrollment methods that integrate pathobiological measures will continue to drive the field toward precision medicine, while mounting clarity on the cardiopulmonary hemodynamic spectrum of risk^34,35^ is likely to widen the range of patients for whom clinical trial enrollment is a consideration. Overall, the trajectory of progress in PAH clinically is exemplar among cardiopulmonary diseases, and ongoing efforts continuing this line of progress appear well-suited to address important knowledge gaps that persist in order to meet patient’s expectations of treatment efficacy and improve outcome further.

## CONFLICTS OF INTEREST

Dr. Maron is a consultant for Actelion Pharmaceuticals, and is an inventor on a US patent 9,605,047 and US provisional patent applications 24622 and 61/99,754.
